# Dirty necrosis in renal cell carcinoma is associated with NETosis and systemic inflammation

**DOI:** 10.1002/cam4.5249

**Published:** 2022-09-20

**Authors:** Takashi Kuroe, Reiko Watanabe, Ryo Morisue, Saori Miyazaki, Motohiro Kojima, Shawhay Charles Murata, Tokiko Nakai, Tetsuro Taki, Shingo Sakashita, Naoya Sakamoto, Nobuaki Matsubara, Hitoshi Masuda, Tetsuo Ushiku, Genichiro Ishii

**Affiliations:** ^1^ Department of Pathology and Clinical Laboratories National Cancer Center Hospital East Kashiwa Chiba Japan; ^2^ Department of Pathology, Graduate School of Medicine The University of Tokyo Tokyo Japan; ^3^ Division of Pathology, Exploratory Oncology Research and Clinical Trial Center National Cancer Center Kashiwa Chiba Japan; ^4^ Department of Hepatobiliary and Pancreatic Surgery National Cancer Center Hospital East Kashiwa Chiba Japan; ^5^ Department of Medical Oncology National Cancer Center Hospital East Kashiwa Chiba Japan; ^6^ Department of Urology National Cancer Center Hospital East Kashiwa Chiba Japan; ^7^ Division of Innovative Pathology and Laboratory Medicine, Exploratory Oncology Research and Clinical Trial Center National Cancer Center Kashiwa Chiba Japan

**Keywords:** dirty necrosis, NETosis, neutrophil extracellular traps, systemic inflammation, tumor necrosis

## Abstract

**Aim:**

Dirty necrosis (DN) in renal cell carcinoma (RCC) is morphologically characterized by abundant neutrophil infiltration and has significant potential as an unfavorable prognostic indicator. This study aimed to analyze the pathological and biological features of DN.

**Materials and Methods:**

A total of 81 RCC tumors, including 33 cases of DN and 48 cases of tumor necrosis without DN features (ghost necrosis [GN]), were enrolled in this study. We compared the number of neutrophils; the activation of cell death pathways, including ferroptosis, NETosis, and apoptosis; the rate of epithelial‐mesenchymal transition (EMT); and proliferation status using immunohistochemistry. We further assessed the effect of the necrosis type on systemic inflammation.

**Results:**

DN tumors had a significantly higher number of neutrophils in both areas around the necrotic foci and far from the necrotic foci. Ferroptosis status did not differ between DN and GN; however, DN tumors had significantly larger areas exhibiting cell detritus with neutrophil extracellular traps (NETs) detected by citrullinated histone H3 (citH3) than GN tumors. DN tumors also had more apoptotic cells within areas around the necrotic foci. There was no significant difference between the EMT and proliferation status between DN and GN groups. Systemic inflammation markers including C‐reactive protein (CRP), CRP‐to‐albumin ratio (CRP/Alb), platelet‐to‐lymphocyte ratio (PLR), and hemoglobin were significantly higher in patients with DN. In addition, some of these inflammation markers (CRP/Alb and PLR) significantly decreased after surgery.

**Conclusions:**

DN in RCC is characterized by NETs production and systemic inflammation.

## Graphical abstract

Dirty necrosis (DN) in renal cell carcinoma (RCC) is morphologically characterized by abundant neutrophil infiltration and has significant potential as an unfavorable prognostic indicator. In addition, DN in RCC is characterized by neutrophil extracellular traps production and systemic inflammation.

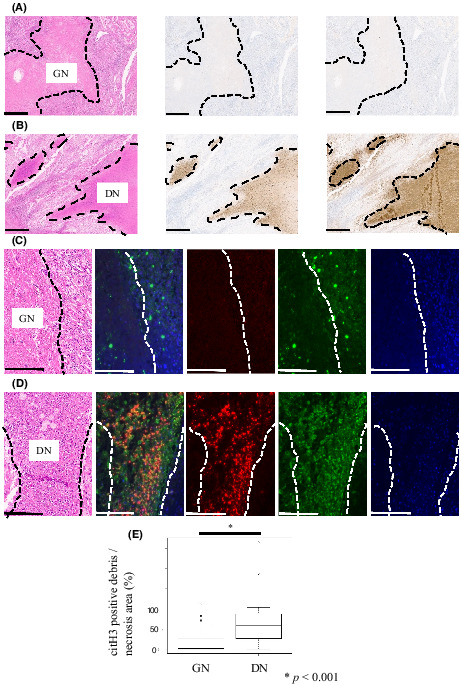

## 1 INTRODUCTION

Tumor necrosis (TN) is one of the important pathological prognostic factors in many types of cancers, including breast, brain (glioblastoma), lung, pancreas, and renal cell carcinoma (RCC).^1^, ^2^, ^3^, ^4^, ^5^

In RCC, we previously showed that there were two subtypes of TN: dirty necrosis (DN) and ghost necrosis (GN),^6^ which showed different clinicopathological features. DN in RCC is characterized by neutrophil infiltration and cell detritus, providing further unfavorable prognostic information beyond the presence of GN. Several studies have investigated the formation of TN; however, its exact pathogenesis remains unclear. Necrosis can be caused by a variety of factors, including ischemia, hypoxia, nutrient starvation, and the immune response.^7^, ^8^, ^9^

Recently, tumor‐associated neutrophils (TANs) have been found to modulate tumor progression.^10^, ^11^, ^12^ However, the details of TANs function or crosstalk with cancer cells, tumor‐infiltrating lymphocytes, and tumor associated macrophages remain poorly understood.

In terms of the relationship between neutrophils and TN, in glioblastoma, neutrophil‐induced ferroptosis promotes TN in tumor progression,^13^ and these neutrophil‐rich necrotic foci are morphologically similar to DN foci in RCC. NETosis is a form of neutrophil‐specific cell death related to protection against infection.^14^, ^15^, ^16^ In this process, activated neutrophils expel their DNA and intracellular contents, such as myeloperoxidase (MPO), a web‐like structure which forms neutrophil extracellular traps (NETs). Recently, many researchers have highlighted the role of NETs in tumor progression and metastasis.^17^, ^18^, ^19^, ^20^

In this study, we focused on how ferroptosis and NETosis affect DN formation. Moreover, we investigated whether TN affects the clinical status in terms of systemic inflammation, and subsequently revealed the difference between DN and GN.

## 2 MATERIALS AND METHODS

### 2.1 Case selection

We enrolled 81 cases of surgically resected RCCs with TN at our institution between January 2011 and September 2020. There were 58 cases of clear cell RCC (ccRCC), 18 cases of papillary RCC (pRCC), and 5 cases of chromophobe RCC (Table S1).

For each case, information on the following clinicopathological features was extracted from medical and pathological records: age, sex, tumor location, tumor size, histological subtypes, pT staging category, WHO/ISUP grade, sarcomatoid differentiation, microvascular invasion, renal vein thrombus, and distant metastases (found on the date of operation). We further extracted patient laboratory data, such as C‐reactive protein (CRP), neutrophil‐to‐lymphocyte ratio, CRP‐to‐albumin ratio (CRP/Alb), platelet‐to‐lymphocyte ratio (PLR), and hemoglobin (Hb). WHO/ISUP grade was applied to ccRCC and pRCC, but not to chromophobe RCC. Pre‐ and postoperative comparisons of systemic inflammation markers were applied to cases without distant metastasis at the date of surgery. Preoperative markers were measured just before the operation and postoperative markers were measured at least 3 months after the operation. All specimens were collected after obtaining comprehensive written informed consent from the patients. This study was approved by the institutional review board of the National Cancer Center (IRB number: 2019‐217).

### 2.2 Subclassification of TN

Hematoxylin and eosin (H&E)‐stained slides with the largest necrotic foci were reviewed by the observers, and TN was classified as one of two subtypes: DN and GN. Both DN and GN were defined according to our previous report.^6^ First, DN was characterized by abundant neutrophil infiltration and cell detritus. Second, GN is characterized by eosinophilic ghost cells without features of DN.

### 2.3 Immunohistochemical analysis

All surgical specimens were fixed in 10% formalin and embedded in paraffin. The tumors were sliced at approximately 5–10 mm intervals, and serial 4‐μm sections were used for immunohistochemical staining. The primary antibodies used in this study are summarized in Table S2. We used the Benchmark ULTRA automated staining system (Roche/Ventana Medical Systems).

### 2.4 Evaluation of immunohistochemistry

The number of neutrophils was counted by detecting CD66b‐positive inflammatory cells (cytoplasm) in four randomly selected spots in both areas around the necrotic foci and far from the necrotic foci, and the average of their four spots was calculated. GPX4 status was classified into three patterns within areas around the necrotic foci; “increased,” “decreased,” and “unchanged,” based on the relative cytoplasmic expression in cancer cells far from the necrotic foci. For anti‐histone H3 (citH3), we detected anti‐citH3‐positive cell detritus within necrotic foci and calculated their area. Aperio AT2 (Aperio ImageScope) was used to calculate the area of the digital slides. For ZEB1, nuclear staining above 10% of viable cancer cells was considered positive. The number of apoptotic cells was counted by detecting cleaved caspase 3‐positive cancer cells (cytoplasm) in the hotspot. Proliferation status was assessed by counting geminin‐positive cancer cells (nuclei) in the hotspot. Furthermore, ‘Areas around the necrotic foci’ was defined as less than 330 μm from the edge of necrotic foci. ‘Areas far from the necrotic foci’ was defined as a viable cancer areas at least 330 μm from the necrotic foci.

### 2.5 Fluorescent multiplex immunohistochemistry

Fluorescent multiplex immunohistochemistry (FMIHC) was performed with the Tyramide Signal Amplification (PerkinElmer) system using an Opal IHC kit (Akoya Biosciences, Inc.), according to the manufacturer's instructions, as previously described.^21^ The antibodies used in this procedure included MPO (A039829‐2; Dako) and anti‐histone H3 (citrulline R2 + R8 + R17) (ab5103; Abcam). We used a fluorescence microscope (Keyence; BZ‐9000) to capture images (Keyence BZ‐II Analyzer Ver 1.41). FMIHC was performed on representative slides containing DN or GN and the co‐expression of MPO and anti‐citH3 was observed in the cell detritus of necrotic foci.

### 2.6 Statistical analysis

Comparisons among clinicopathological features, necrosis types, and immunohistochemistry results were evaluated using Fisher's exact test and Mann–Whitney *U*‐test. The correlation between CD66b‐positive neutrophils and apoptotic cell numbers was evaluated using the Spearman rank correlation coefficient, and difference in pre‐ or postoperative laboratory findings was evaluated using a paired *t*‐test. All statistical analyses were performed using EZR (Saitama Medical Center, Jichi Medical University, Saitama, Japan), a graphical user interface for R (The R Foundation for Statistical Computing).^22^ *p* values <0.05 were considered statistically significant.

## 3 RESULTS

### 3.1 Clinicopathological characteristics of RCC with DN

Figure S1 shows the characteristic features of RCC with GN and DN. GN is characterized by the presence of ghost cells with eosinophilic cytoplasm without DN features (Figure S1A,B). Fibrosis and hyalinization often coexist in GN tumors (Figure S1C). In contrast, DN tumors exhibit multiple necrotic foci with neutrophil infiltration and cell detritus (Figure S1D–F). We identified 48 cases of GN and 33 cases of DN in 81 patients. DN was significantly associated with WHO/ISUP grade, sarcomatoid differentiation (DN vs. GN: 15% vs. 2%), and distant metastasis (36% vs. 12%). Age, sex, tumor location (left or right), tumor size, histology, and pT staging category were not associated with the necrosis type (Table S1). We could not find significant difference between microvascular invasion or renal vein thrombus and DN in the current study (Table S3). In addition, we identified both GN and DN not only in ccRCC, but also in pRCC.

### 3.2 Comparison of the number of neutrophils between DN and GN

**FIGURE 1:**
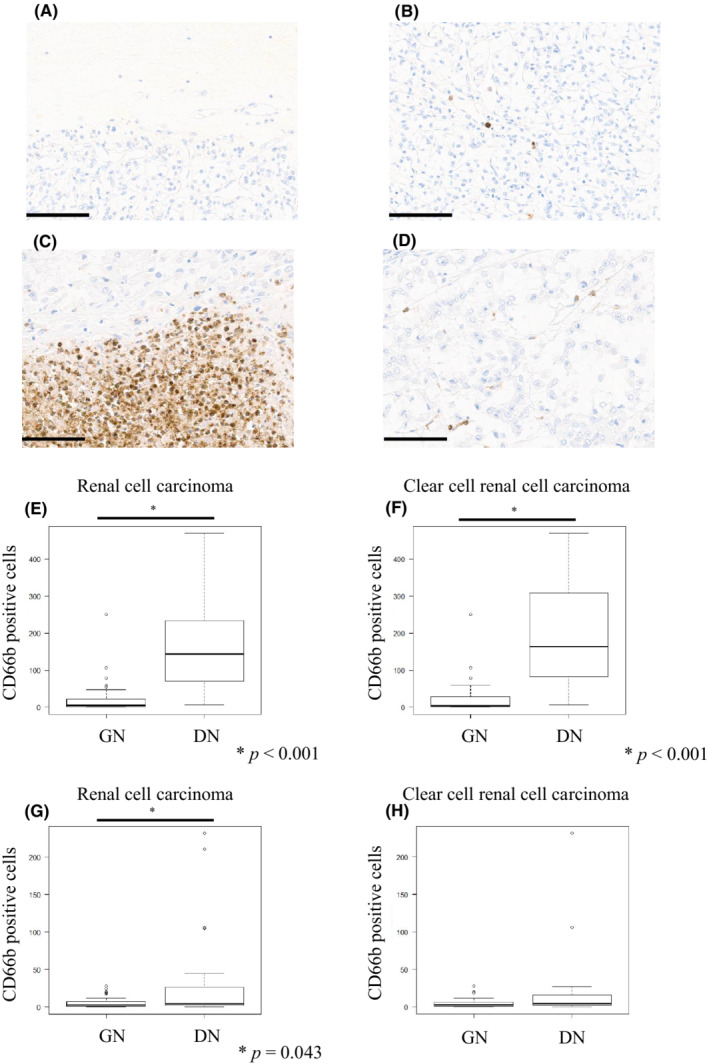
Comparison of the number of CD66b‐positive neutrophils between dirty and ghost necrosis (GN). (A) Representative CD66b staining within areas around the necrotic foci in GN tumors. (B) Representative CD66b staining within areas far from the necrotic foci in GN tumors. (C) Representative CD66b staining within areas around necrotic foci in dirty necrosis (DN) tumors. (D) Representative CD66b staining within areas far from necrotic foci in DN tumors (scale bars = 100 μm). (E) Comparison of CD66b‐positive cell numbers between GN and DN tumors within areas around the necrotic foci (Mann–Whitney *U*‐test, *p* < 0.001). (F) Comparison of CD66b‐positive cell numbers between GN and DN tumors within areas around the necrotic foci confined to clear cell renal cell carcinoma (ccRCC) (Mann–Whitney *U*‐test, *p* < 0.001). (G) Comparison of CD66b‐positive cell numbers between GN and DN tumors within areas far from necrotic foci (Mann–Whitney *U*‐test, *p* = 0.043). (H) Comparison of CD66b‐positive cell numbers between GN and DN tumors within areas far from the necrotic foci confined to ccRCC (Mann–Whitney *U*‐test, *p* = 0.102).

We used immunohistochemistry (CD66b) to identify the exact neutrophil infiltration. As shown in Figure 1, GN tumors showed very little neutrophil infiltration in both areas around the necrotic foci and far from the necrotic foci (Figure 1A,B). However, DN tumors showed considerable neutrophil infiltration within areas around the necrotic foci (Figure 1C), and a few neutrophils within areas far from the necrotic foci (Figure 1D). Neutrophil infiltration of DN within areas around the necrotic foci was significantly higher than that of GN (*p* < 0.001) (Figure 1E), and was almost equally confined to ccRCC (*p* < 0.001) (Figure 1F). In addition, neutrophil infiltration of DN within areas far from the necrotic foci was also significantly higher than those of GN (*p* = 0.043) (Figure 1G); however, there was no significant difference in DN confined to ccRCC (Figure 1H).

### 3.3 Comparison of ferroptosis status between DN and GN

**FIGURE 2:**
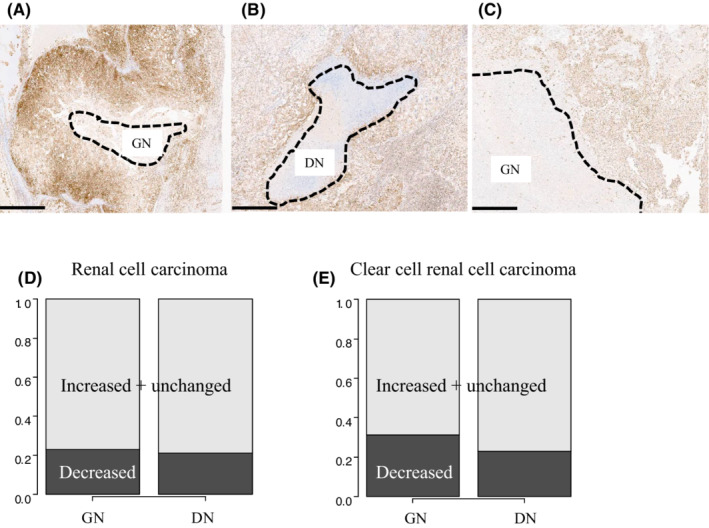
GPX4 expression within the areas around the necrotic foci. (A) Representative decreased GPX4 staining within areas around the necrotic foci. (B) Representative increased GPX4 staining within areas around the necrotic foci. (C) Representative unchanged GPX4 staining within areas around the necrotic foci (scale bars = 300 μm). (D) Comparison of GPX4 staining patterns (decreased vs. increased + unchanged) between ghost necrosis (GN) and dirty necrosis (DN) tumors (Fisher's exact test, *p* = 1). (E) Comparison of GPX4 staining patterns (decreased vs. increased + unchanged) between GN and DN tumors confined to clear cell renal cell carcinoma (Fisher's exact test, *p* = 0.564).

We assessed the GPX4 status of areas around the necrotic foci to determine whether ferroptosis affects DN. As shown in Figure 2, representative GPX4 status was classified into three patterns: decreased (Figure 2A), increased (Figure 2B), and unchanged (Figure 2C). The rate of the decreased status, the characteristic staining pattern for ferroptosis, were almost the same regardless of necrosis type (Figure 2D, GN vs. DN: 23% vs. 21%). We also found similar results in the ccRCC‐limited cohort (Figure 2E, GN vs. DN: 31% vs. 23%).

### 3.4 Comparison of NETosis status between DN and GN

**FIGURE 3:**
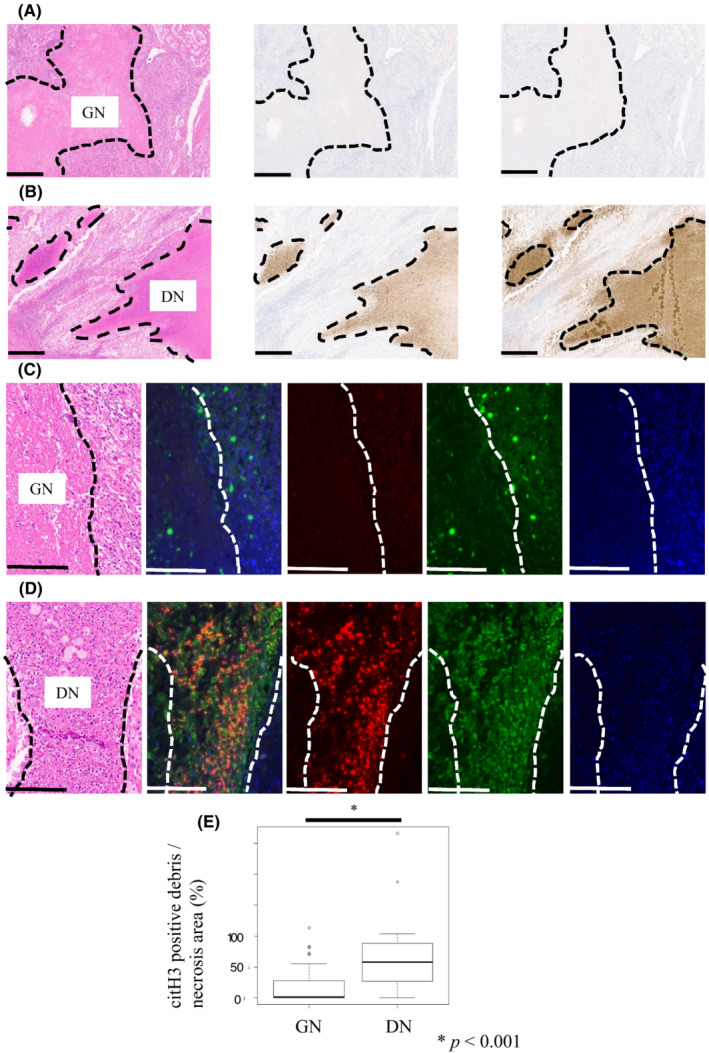
citH3 and myeloperoxidase (MPO) expression in necrotic foci. (A) Representative hematoxylin and eosin (H&E) and immunohistochemical slides in the necrotic foci of ghost necrosis (GN) (left, H&E; middle, citH3; right, MPO). (B) Representative H&E and immunohistochemical slides in the necrotic foci of dirty necrosis (DN) (left, H&E; middle, citH3; right, MPO) (scale bars = 300 μm). (C) Representative H&E and fluorescent multiplex immunohistochemistry (FMIHC) slides in the necrotic foci of GN. The expression of citH3, MPO, and DAPI in the cells is shown in red, green, and blue, respectively. (D) Representative H&E FMIHC slides in the necrotic foci of DN. The expression of citH3, MPO, and DAPI in the cells is shown in red, green, and blue, respectively. FMIHC shows co‐expression of citH3 and MPO, which indicates cell detritus, including neutrophil extracellular traps (NETs) (scale bars = 100 μm). (E) Comparison of citH3‐positive cell detritus/necrotic area between GN and DN tumors (Mann–Whitney *U*‐test, *p* < 0.001).

Figure 3A (left) shows a representative H&E slide of GN. Anti‐citH3‐positive cell detritus was almost absent within the necrotic foci in GN tumors (Figure 3A, middle), and cell detritus in GN was almost negative for MPO (Figure 3A, right). A representative H&E slide of DN is shown in Figure 3B (left). In contrast to GN, anti‐citH3‐positive cell detritus was distinct in DN tumors (Figure 3B, middle), and was also strongly positive for MPO (Figure 3B, right). We performed FMIHC to observe the coexistence of MPO and citH3, and representative images of GN and DN tumors are shown in Figure 3C,D, respectively. As expected, the coexistence of MPO and citH3 was observed in DN tumors, and the area of citH3‐positive cell detritus in DN tumors was larger than that in GN tumors (*p* < 0.001) (Figure 3E).

### 3.5 Comparison of epithelial‐mesenchymal transition status between DN and GN

As shown in Table S1, DN was associated with sarcomatoid differentiation. Therefore, we examined the epithelial‐mesenchymal transition status of cancer cells within both areas around the necrotic foci and far from the necrotic foci, using a ZEB‐1 antibody (Figure S2). Table S4 shows that the frequency of ZEB1‐positive cancer cells was not significantly different between DN and GN tumors, regardless of their location, and was almost equal to that of ccRCC.

### 3.6 Comparison of apoptosis and proliferation status between DN and GN

**FIGURE 4:**
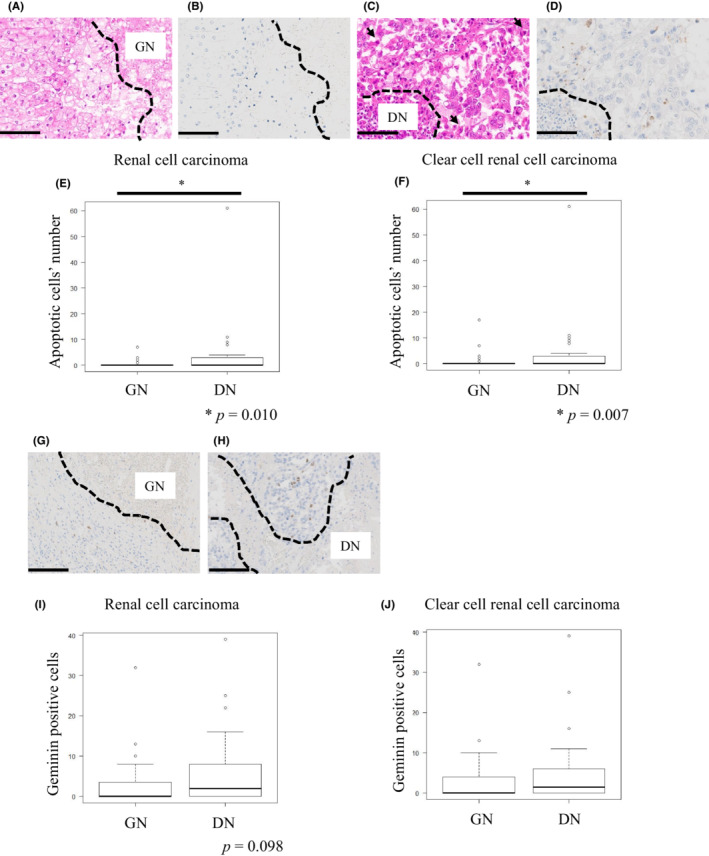
Expression of cleaved caspase 3 and geminin within the areas around the necrotic foci. (A, B) Representative hematoxylin and eosin (H&E) and cleaved caspase 3 staining within areas around the necrotic foci in ghost necrosis (GN) tumors. (C, D) Representative H&E and cleaved caspase 3 staining within areas around necrotic foci in dirty necrosis (DN) tumors. (E) Comparison of apoptotic cell numbers between GN and DN tumors within areas around the necrotic foci (Mann–Whitney *U*‐test, *p* = 0.010). (F) Comparison of numbers of apoptotic cells between GN and DN tumors within areas around the necrotic foci confined to clear cell renal cell carcinoma (ccRCC) (Mann–Whitney *U*‐test, *p* = 0.007). (G, H) Representative geminin slides within areas around the necrotic foci in GN (G) and DN (H) tumors (scale bars = 100 μm). (I) Comparison of geminin‐positive cell numbers between GN and DN tumors within areas around the necrotic foci (Mann–Whitney *U*‐test, *p* = 0.098). (J) Comparison of geminin‐positive cell numbers between GN and DN tumors within areas around the necrotic foci confined to ccRCC (Mann–Whitney *U*‐test, *p* = 0.452).

As shown in Figure 4A–D, cancer cells with DN displayed a significantly higher proportion of cleaved caspase 3‐positive cancer cells within areas around the necrotic foci (*p* = 0.010) (Figure 4E). It was also almost equally confined to ccRCC‐limeted cohort (*p* = 0.007) (Figure 4F). However, there was no significant difference within areas far from the necrotic foci between the DN and GN groups (Figure S3A,B). Furthermore, we assessed the correlation between the number of CD66b‐positive neutrophils and apoptotic cells within areas around the necrotic foci in cases of both GN and DN. There was no significant correlation between GN and DN (Figure S4).

Next, we assessed the proliferation status of cancer cells within both areas around the necrotic foci and far from the necrotic foci using anti‐Geminin antibody (Figure 4G,H). Cancer cells with DN tended to show higher numbers of geminin‐positive cancer cells within areas around the necrotic foci (*p* = 0.098) (Figure 4I); however, no significant difference was observed in ccRCC‐limited cohort (Figure 4J), there was also no significant difference in areas far from the necrotic foci between the GN and DN groups (Figure S3C,D).

### 3.7 Comparison of necrosis types and laboratory findings related to systemic inflammation

**FIGURE 5:**
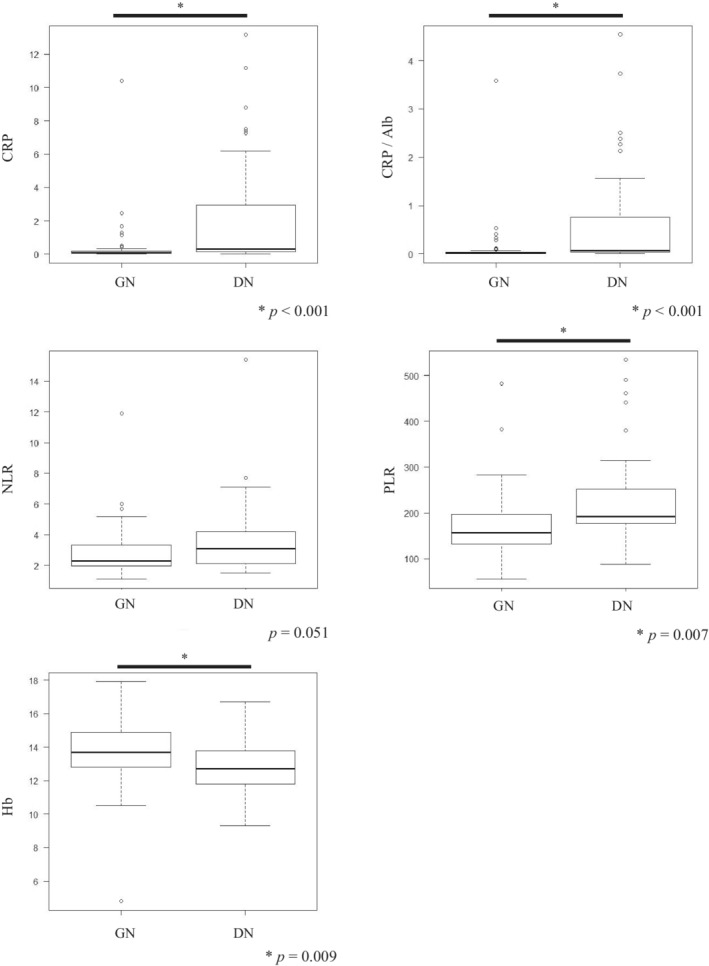
Comparison of necrosis types and laboratory findings related to systemic inflammation. CRP, C‐reactive protein; CRP/Alb, CRP‐to‐albumin ratio; Hb, hemoglobin; NLR, neutrophil‐to‐lymphocyte ratio; PLR, platelet‐to‐lymphocyte ratio.

**FIGURE 6:**
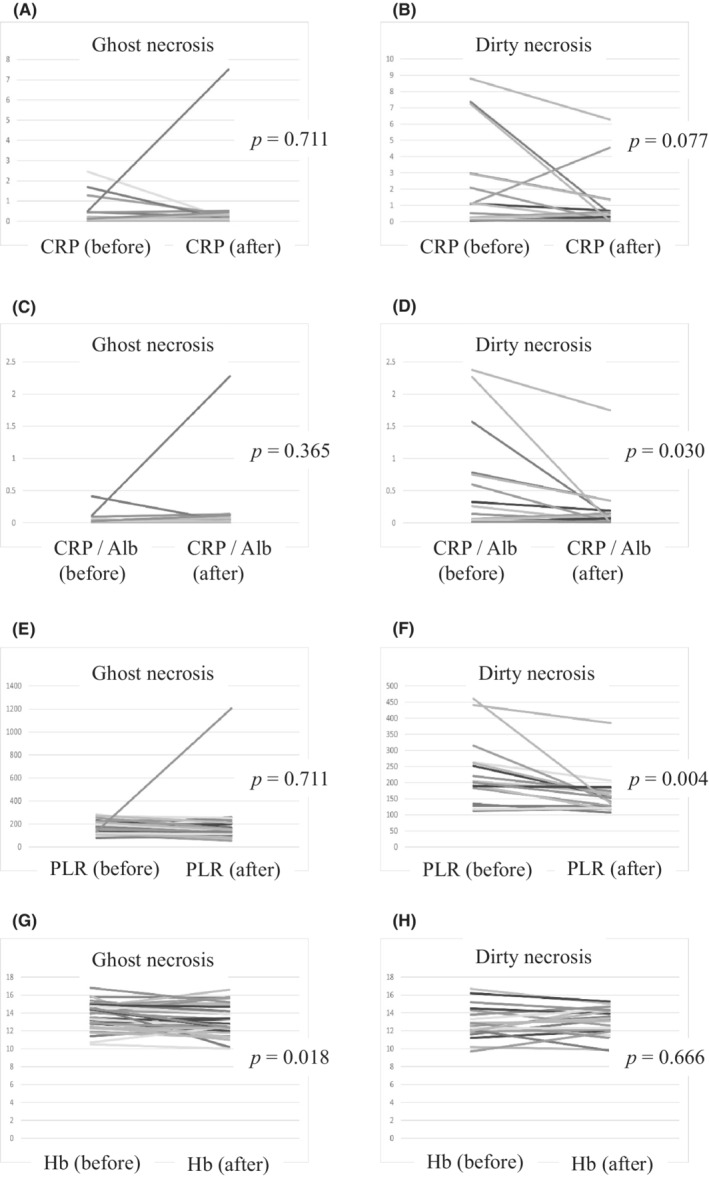
Laboratory findings related to systemic inflammation before and after surgery. CRP, C‐reactive protein; CRP/Alb, CRP‐to‐albumin ratio; Hb, hemoglobin; PLR, platelet‐to‐lymphocyte ratio.

NETosis has been implicated in tumor inflammation; however, little is known regarding the clinicopathological association between NETosis and systemic inflammation.^23^ As shown in Figure 5, multiple laboratory findings (CRP, CRP/Alb, PLR) revealed that DN tumors were associated with systemic inflammation and anemia of chronic disorder (Hb). Furthermore, it is worth noting that two laboratory findings (CRP/Alb and PLR) significantly decreased postoperatively in DN tumors (*p* = 0.030 and 0.004, respectively) (Figure 6D,F), while CRP levels also tended to decrease (*p* = 0.077). However, GN tumors did not change before or after surgery (Figure 6C,E). Incidentally, we found no significant difference in tumor size depending on the necrosis type (Table S1). In contrast, the Hb level was significantly decreased in GN tumors postoperatively (*p* = 0.018) (Figure 6G), showing no change in DN tumors (Figure 6H).

## 4 DISCUSSION

This study is the first to report that DN in RCC is associated with NETosis. We further identified that many systemic inflammation markers were significantly higher in DN tumors and that some of the markers significantly decreased after surgery. This indicated that DN in RCC is associated with systemic inflammation.

Previous reports have indicated that TN is generally caused by cancer cell death. However, the present study indicates that NETosis underlies DN, and further shows the important implication of investigating not only cancer cell death, but also neutrophil death. Our results showing the association between DN with high NETs production supports previous reports that NETs may exert a protective effect on cancer cells from attack by T‐lymphocytes or NK cells, and that NETs could capture tumor cells to promote distant metastasis.^23^, ^24^ Prior studies in glioblastoma have shown that neutrophil‐induced ferroptosis promoted TN in tumor progression; however, the current study revealed that ferroptosis did not affect DN in RCC.^13^

We also investigated DN cancer cells within areas around the necrotic foci. Although DN cancer cells showed a higher proportion of sarcomatoid differentiation, immunohistochemical staining for ZEB1 revealed no significant difference between DN and GN tumors (Table S4).

We found that DN cancer cells showed a higher number of apoptotic cells within areas around the necrotic foci than GN cancer cells (Figure 4). In addition, we found that DN cancer cells tended to have a higher proliferation status than GN cancer cells (Figure 4). These results indicate the biological difference of cancer cells with respect to the necrosis type, and suggest that this difference may be a reflection of the high malignant potential in DN tumors, such as the phoenix rising pathway activated by apoptotic cells, and a high proliferation rate.^25^, ^26^ Our findings, that DN tumors showed a high proliferation rate and produced a lot of NETs, is consistent with a previous study indicating that NETs could also support tumor growth.^27^

Finally, we investigated the contribution of DN to systemic inflammation. We found that various inflammation markers were significantly higher in DN tumors than in GN tumors. In addition, a comparison of inflammation markers before and after surgery revealed that some were significantly more lowly expressed after surgery in patients with DN (Figure 6). This indicates that DN is closely associated with systemic inflammation, although we found no significant correlation between tumor volume and the type of necrosis. Some previous studies have shown that NETs can promote tumor venous embolism by activating platelets; however, relatively few reports have investigated how NETosis affects systemic inflammation in human cancers.^23^ On the other hand, a few studies with regard to sepsis have shown the relationship between NETosis and systemic inflammation, and Valentina Poli et al. showed that inhibiting NETs production also reduced systemic inflammation.^28^, ^29^ Although we were not able to directly prove the effect of NETosis on systemic inflammation, our results imply this relationship through the comparison of inflammation markers. Taken together, DN tumors may induce inflammation both locally and systemically.

This study has some limitations. First, although we showed the comparison of inflammation markers before and after surgery, we need to further investigate the detailed classification of clinical stages to apply these research findings to clinical medicine. Second, it would be desirable to prepare more uniform cohort regarding histology such as ccRCC and pRCC independently; thus we need to accumulate much more GN or DN tumors. Third, this is a TN study of only one organ, the kidney; thus, further investigations in other types of cancer are needed to fully validate these findings. Fourth, we could not elucidate the molecular mechanisms underlying DN, and therefore, mouse model of TN needs to be established for further investigations.

In conclusion, our pathological findings suggest that TANs and NETosis may be two key factors driving DN and leading the clinicopathological difference between DN and GN. In addition, DN is not only related to a high malignant potential and poor prognosis, but also to systemic inflammation, which implies that tumors exhibiting DN require more careful patient management and therapy.

## AUTHOR CONTRIBUTIONS

**Takashi Kuroe:** Conceptualization (equal); data curation (lead); formal analysis (lead); investigation (lead); writing – original draft (lead); writing – review and editing (lead). **Reiko Watanabe:** Data curation (supporting); writing – review and editing (supporting). **Ryo Morisue:** Data curation (supporting); writing – review and editing (supporting). **Saori Miyazaki:** Writing – review and editing (supporting). **Motohiro Kojima:** Data curation (supporting); writing – review and editing (supporting). **Shawhay Charles Murata:** Writing – review and editing (supporting). **Tokiko Nakai:** Writing – review and editing (supporting). **Tetsuro Taki:** Writing – review and editing (supporting). **Shingo Sakashita:** Writing – review and editing (supporting). **Naoya Sakamoto:** Writing – review and editing (supporting). **Nobuaki Matsubara:** Writing – review and editing (supporting). **Hitoshi Masuda:** Writing – review and editing (supporting). **Tetsuo Ushiku:** Writing – review and editing (supporting). **Genichiro Ishii:** Conceptualization (equal); investigation (supporting); project administration (lead); resources (lead); supervision (lead); writing – review and editing (supporting).

## FUNDING INFORMATION

This work was supported in part by National cancer center research fund (2020‐A‐9) and JSPS KAKENHI (21H02931).

## CONFLICT OF INTEREST

The authors have no conflict of interest to disclose.

## ETHICS APPROVAL STATEMENT

This study was approved by the Institutional Review Board of the National Cancer Center (IRB number: 2019‐217).

## PATIENT CONSENT STATEMENT

All specimens were collected after obtaining comprehensive written informed consent from the patients.

## Supporting information


Table S1

Table S2

Table S3

Table S4

Figure S1

Figure S2

Figure S3

Figure S4

## Data Availability

The datasets generated or analyzed during the current study are available from the corresponding author on reasonable request.
