# Supplementary material for: Dirty necrosis in renal cell carcinoma is associated with NETosis and systemic inflammation
Source: Cancer Med. 2022 Sep 20;12(4):4557–67. doi: 10.1002/cam4.5249 (PMC9972113; doi:10.1002/cam4.5249)
Supplement: Supplementary file 1 — Table S1 [file CAM4-12-4557-s003.pptx]

## Slide 1
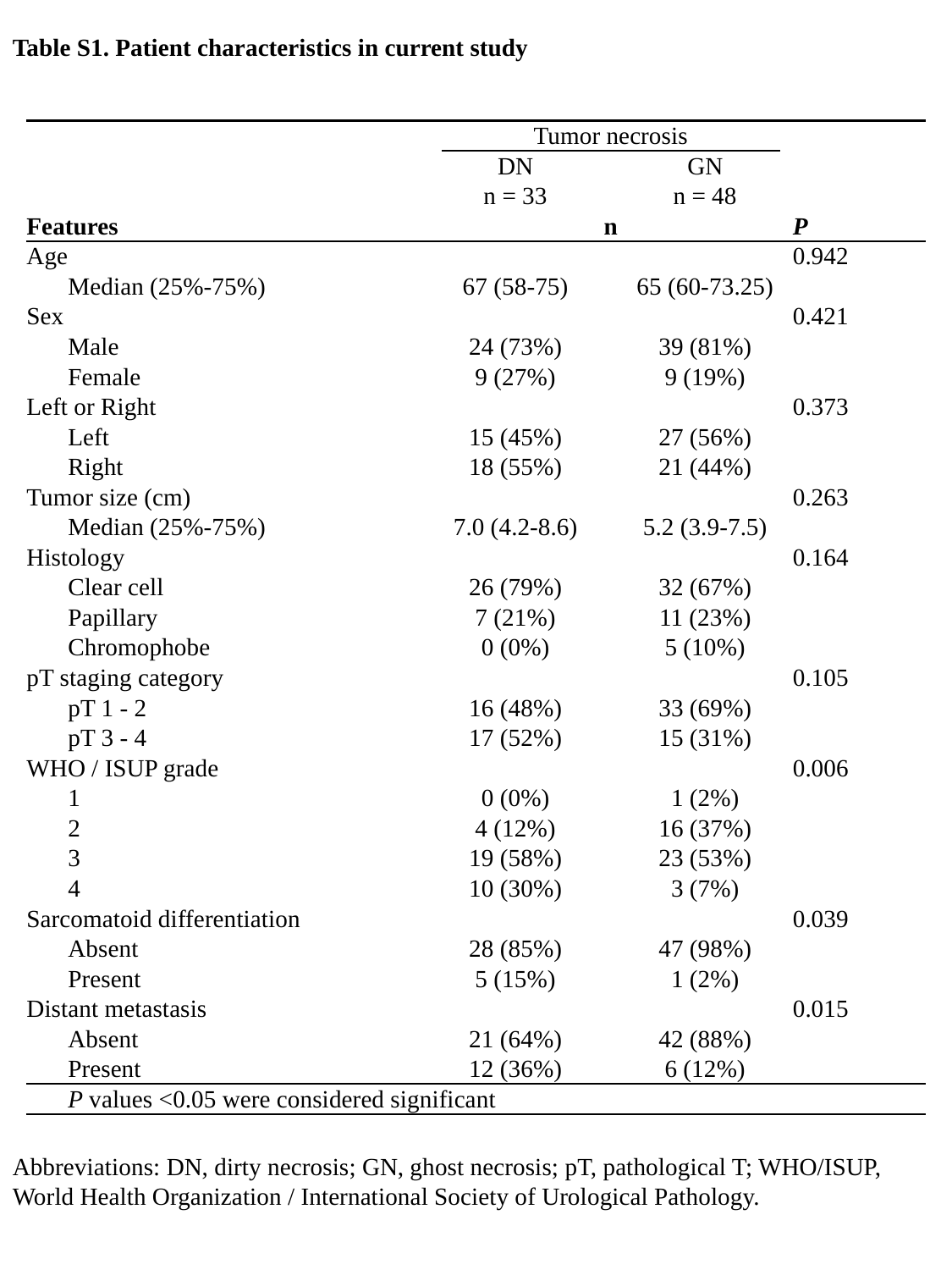

Table S1. Patient characteristics in current study
| | | | | | | | |
| --- | --- | --- | --- | --- | --- | --- | --- |
| | | | Tumor necrosis | | | | |
| | | | DN | | GN | | |
| | | | n = 33 | | n = 48 | | |
| Features | | | n | | | | P |
| Age | | | | | | | 0.942 |
| | Median (25%-75%) | | 67 (58-75) | | 65 (60-73.25) | | |
| Sex | | | | | | | 0.421 |
| | Male | | 24 (73%) | | 39 (81%) | | |
| | Female | | 9 (27%) | | 9 (19%) | | |
| Left or Right | | | | | | | 0.373 |
| | Left | | 15 (45%) | | 27 (56%) | | |
| | Right | | 18 (55%) | | 21 (44%) | | |
| Tumor size (cm) | | | | | | | 0.263 |
| | Median (25%-75%) | | 7.0 (4.2-8.6) | | 5.2 (3.9-7.5) | | |
| Histology | | | | | | | 0.164 |
| | Clear cell | | 26 (79%) | | 32 (67%) | | |
| | Papillary | | 7 (21%) | | 11 (23%) | | |
| | Chromophobe | | 0 (0%) | | 5 (10%) | | |
| pT staging category | | | | | | | 0.105 |
| | pT 1 - 2 | | 16 (48%) | | 33 (69%) | | |
| | pT 3 - 4 | | 17 (52%) | | 15 (31%) | | |
| WHO / ISUP grade | | | | | | | 0.006 |
| | 1 | | 0 (0%) | | 1 (2%) | | |
| | 2 | | 4 (12%) | | 16 (37%) | | |
| | 3 | | 19 (58%) | | 23 (53%) | | |
| | 4 | | 10 (30%) | | 3 (7%) | | |
| Sarcomatoid differentiation | | | | | | | 0.039 |
| | Absent | | 28 (85%) | | 47 (98%) | | |
| | Present | | 5 (15%) | | 1 (2%) | | |
| Distant metastasis | | | | | | | 0.015 |
| | Absent | | 21 (64%) | | 42 (88%) | | |
| | Present | | 12 (36%) | | 6 (12%) | | |
| | P values <0.05 were considered significant | | | | | | |
| | | | | | | | |
Abbreviations: DN, dirty necrosis; GN, ghost necrosis; pT, pathological T; WHO/ISUP, World Health Organization / International Society of Urological Pathology.
