# Supplementary material for: Dirty necrosis in renal cell carcinoma is associated with NETosis and systemic inflammation
Source: Cancer Med. 2022 Sep 20;12(4):4557–67. doi: 10.1002/cam4.5249 (PMC9972113; doi:10.1002/cam4.5249)
Supplement: Supplementary file 2 — Table S2 [file CAM4-12-4557-s008.pptx]

## Slide 1
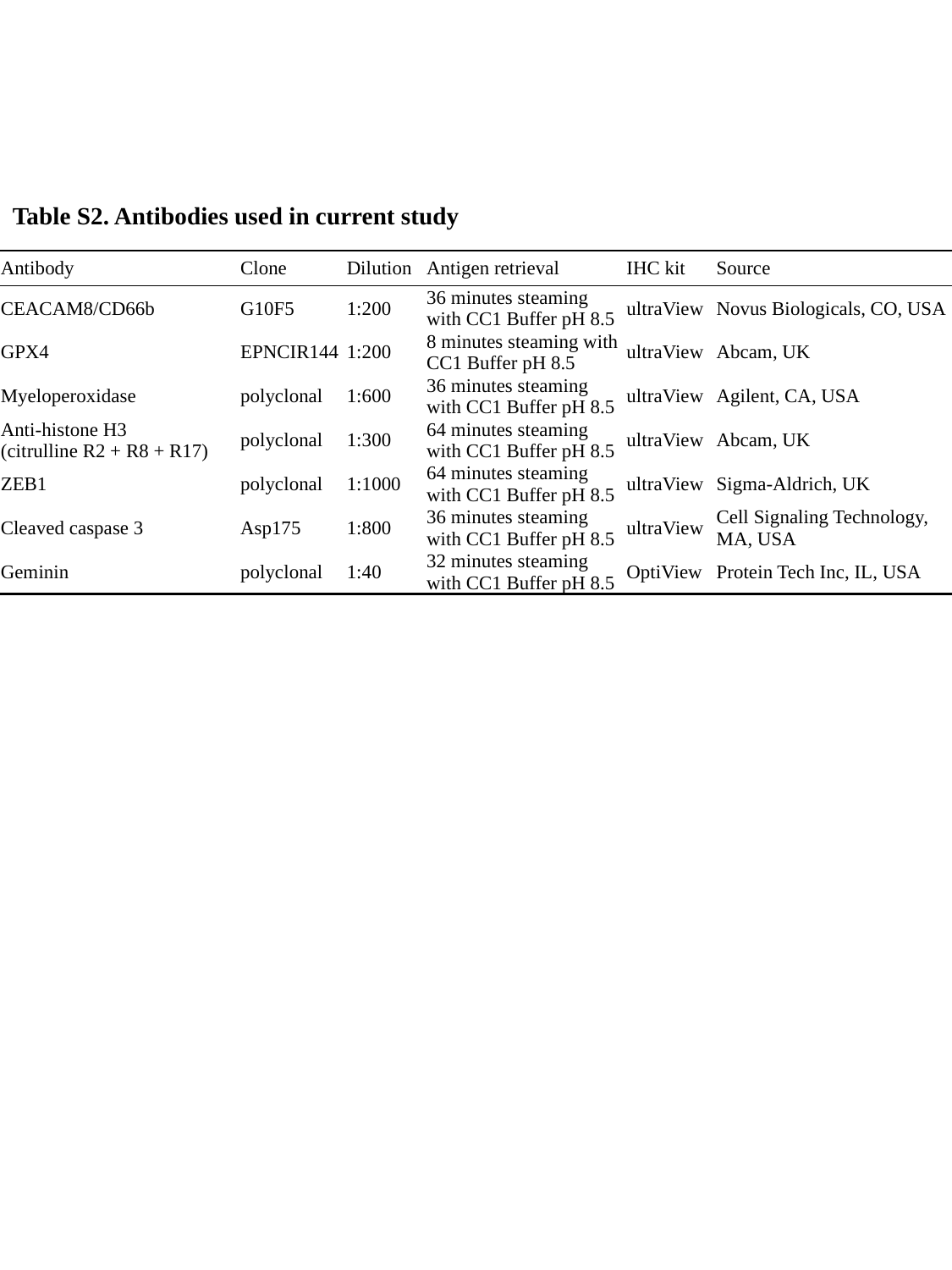

Table S2. Antibodies used in current study
| | | | | | |
| --- | --- | --- | --- | --- | --- |
| Antibody | Clone | Dilution | Antigen retrieval | IHC kit | Source |
| CEACAM8/CD66b | G10F5 | 1:200 | 36 minutes steaming with CC1 Buffer pH 8.5 | ultraView | Novus Biologicals, CO, USA |
| GPX4 | EPNCIR144 | 1:200 | 8 minutes steaming with CC1 Buffer pH 8.5 | ultraView | Abcam, UK |
| Myeloperoxidase | polyclonal | 1:600 | 36 minutes steaming with CC1 Buffer pH 8.5 | ultraView | Agilent, CA, USA |
| Anti-histone H3 (citrulline R2 + R8 + R17) | polyclonal | 1:300 | 64 minutes steaming with CC1 Buffer pH 8.5 | ultraView | Abcam, UK |
| ZEB1 | polyclonal | 1:1000 | 64 minutes steaming with CC1 Buffer pH 8.5 | ultraView | Sigma-Aldrich, UK |
| Cleaved caspase 3 | Asp175 | 1:800 | 36 minutes steaming with CC1 Buffer pH 8.5 | ultraView | Cell Signaling Technology, MA, USA |
| Geminin | polyclonal | 1:40 | 32 minutes steaming with CC1 Buffer pH 8.5 | OptiView | Protein Tech Inc, IL, USA |
| | | | | | |
