# Supplementary material for: Dirty necrosis in renal cell carcinoma is associated with NETosis and systemic inflammation
Source: Cancer Med. 2022 Sep 20;12(4):4557–67. doi: 10.1002/cam4.5249 (PMC9972113; doi:10.1002/cam4.5249)
Supplement: Supplementary file 3 — Table S3 [file CAM4-12-4557-s004.pptx]

## Slide 1
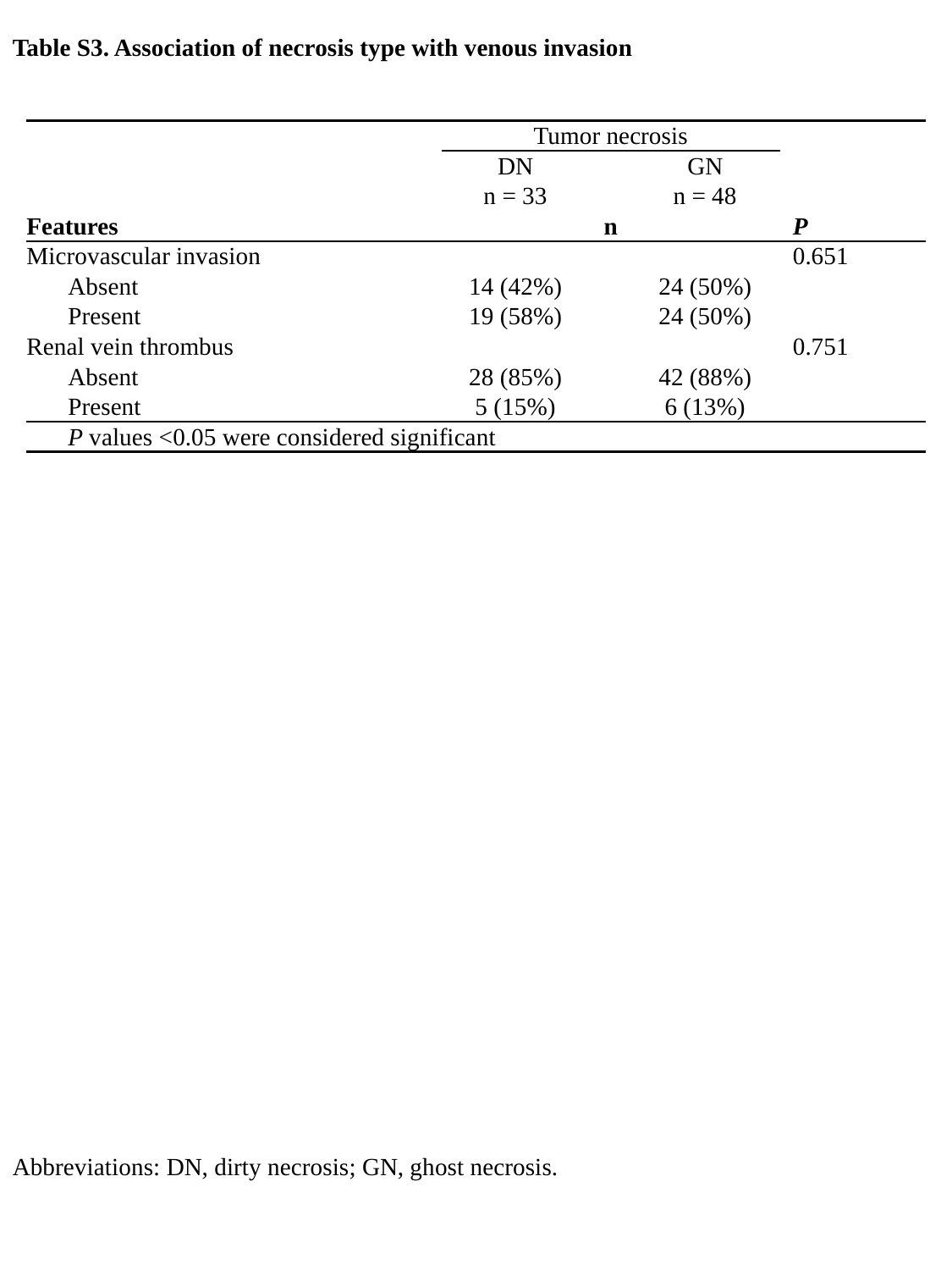

Table S3. Association of necrosis type with venous invasion
| | | | | | | | |
| --- | --- | --- | --- | --- | --- | --- | --- |
| | | | Tumor necrosis | | | | |
| | | | DN | | GN | | |
| | | | n = 33 | | n = 48 | | |
| Features | | | n | | | | P |
| Microvascular invasion | | | | | | | 0.651 |
| | Absent | | 14 (42%) | | 24 (50%) | | |
| | Present | | 19 (58%) | | 24 (50%) | | |
| Renal vein thrombus | | | | | | | 0.751 |
| | Absent | | 28 (85%) | | 42 (88%) | | |
| | Present | | 5 (15%) | | 6 (13%) | | |
| | P values <0.05 were considered significant | | | | | | |
| | | | | | | | |
Abbreviations: DN, dirty necrosis; GN, ghost necrosis.
