# Supplementary material for: Dirty necrosis in renal cell carcinoma is associated with NETosis and systemic inflammation
Source: Cancer Med. 2022 Sep 20;12(4):4557–67. doi: 10.1002/cam4.5249 (PMC9972113; doi:10.1002/cam4.5249)
Supplement: Supplementary file 4 — Table S4 [file CAM4-12-4557-s007.pptx]

## Slide 1
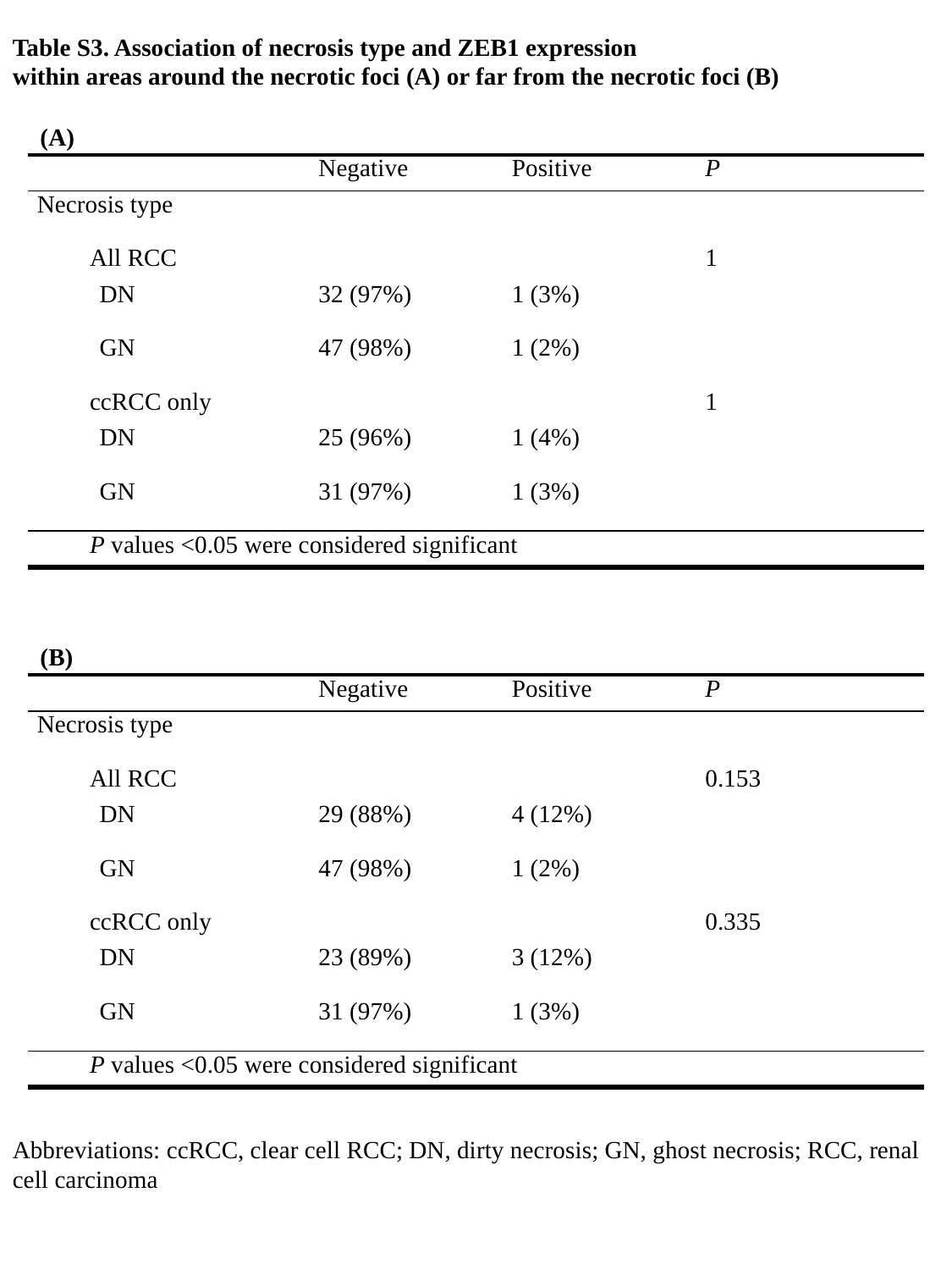

Table S3. Association of necrosis type and ZEB1 expression
within areas around the necrotic foci (A) or far from the necrotic foci (B)
(A)
| | | Negative | Positive | P |
| --- | --- | --- | --- | --- |
| Necrosis type | | | | |
| | All RCC | | | 1 |
| | DN | 32 (97%) | 1 (3%) | |
| | GN | 47 (98%) | 1 (2%) | |
| | ccRCC only | | | 1 |
| | DN | 25 (96%) | 1 (4%) | |
| | GN | 31 (97%) | 1 (3%) | |
| | P values <0.05 were considered significant | | | |
(B)
| | | Negative | Positive | P |
| --- | --- | --- | --- | --- |
| Necrosis type | | | | |
| | All RCC | | | 0.153 |
| | DN | 29 (88%) | 4 (12%) | |
| | GN | 47 (98%) | 1 (2%) | |
| | ccRCC only | | | 0.335 |
| | DN | 23 (89%) | 3 (12%) | |
| | GN | 31 (97%) | 1 (3%) | |
| | P values <0.05 were considered significant | | | |
Abbreviations: ccRCC, clear cell RCC; DN, dirty necrosis; GN, ghost necrosis; RCC, renal cell carcinoma
