# Supplementary figures and images for: Dirty necrosis in renal cell carcinoma is associated with NETosis and systemic inflammation
Source: Cancer Med. 2022 Sep 20;12(4):4557–67. doi: 10.1002/cam4.5249 (PMC9972113; doi:10.1002/cam4.5249)

## Slide 1
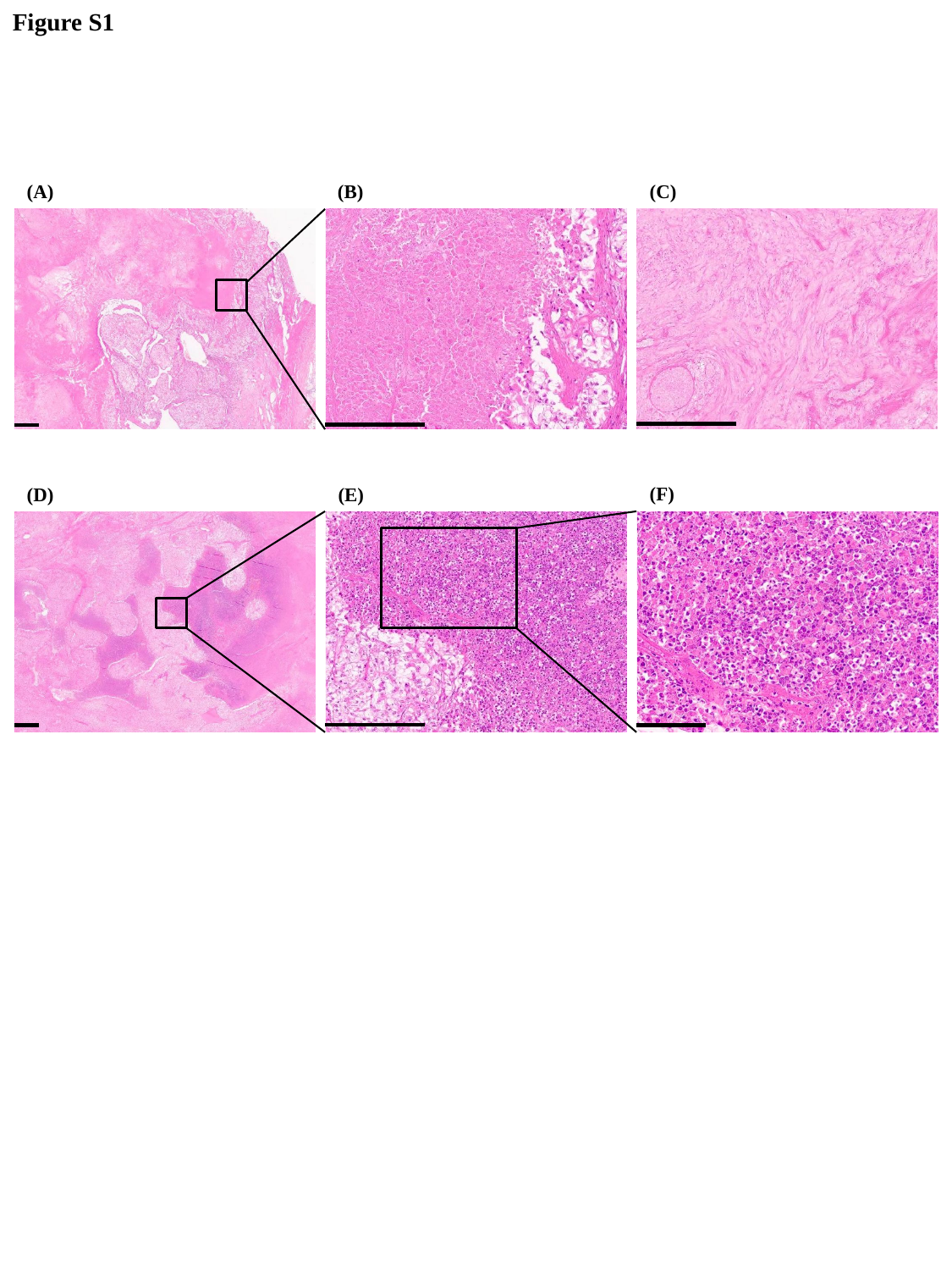

Figure S1
(A)
(B)
(C)
(F)
(D)
(E)

Supplement: Supplementary file 5 — Figure S1 [file CAM4-12-4557-s002.pptx]

## Slide 1
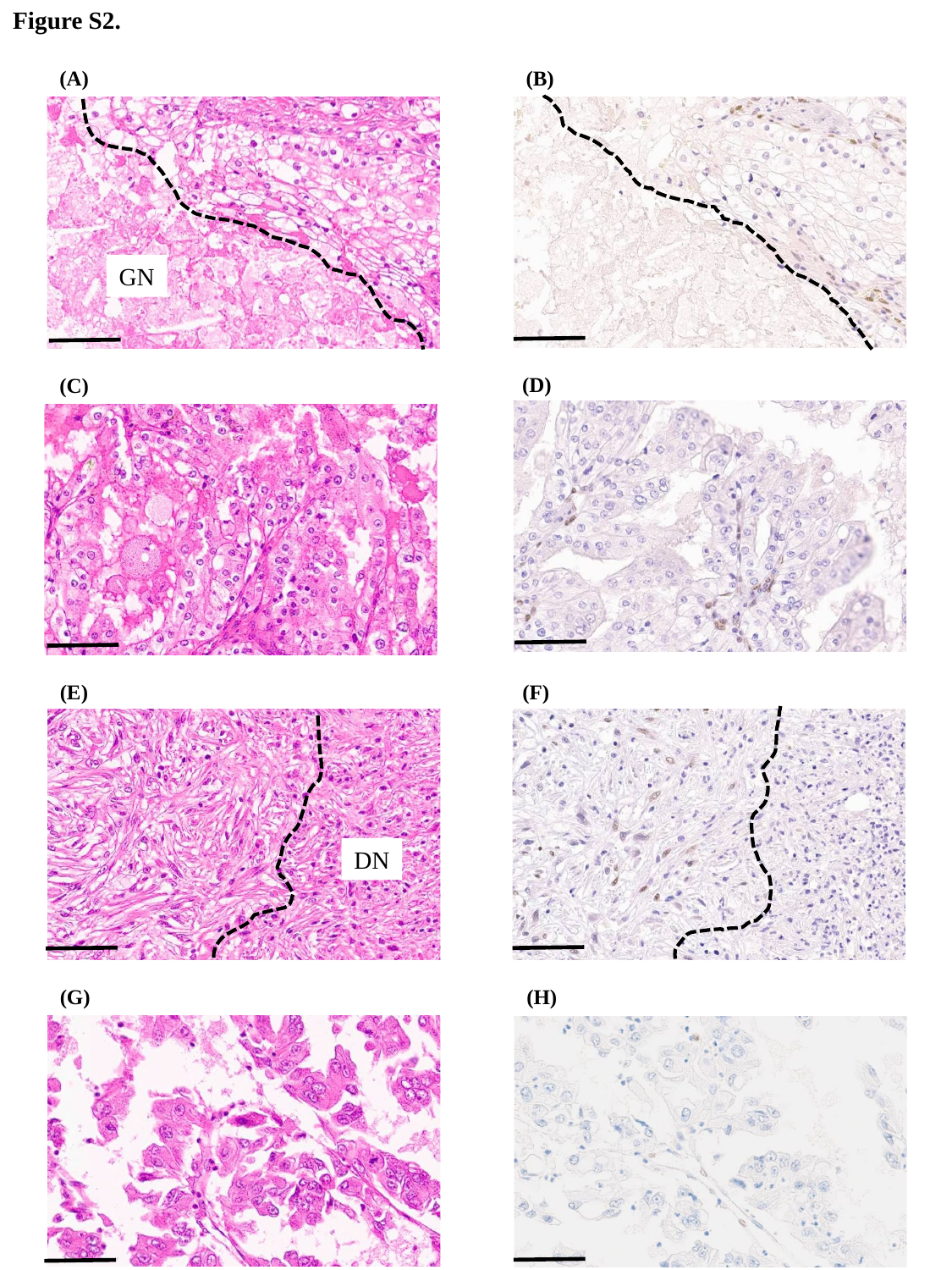

Figure S2.
(A)
(B)
(D)
(C)
GN
(E)
(F)
(G)
(H)
DN

Supplement: Supplementary file 6 — Figure S2 [file CAM4-12-4557-s001.pptx]
