# Supplementary material for: Dirty necrosis in renal cell carcinoma is associated with NETosis and systemic inflammation
Source: Cancer Med. 2022 Sep 20;12(4):4557–67. doi: 10.1002/cam4.5249 (PMC9972113; doi:10.1002/cam4.5249)
Supplement: Supplementary file 7 — Figure S3 [file CAM4-12-4557-s006.pptx]

## Slide 1
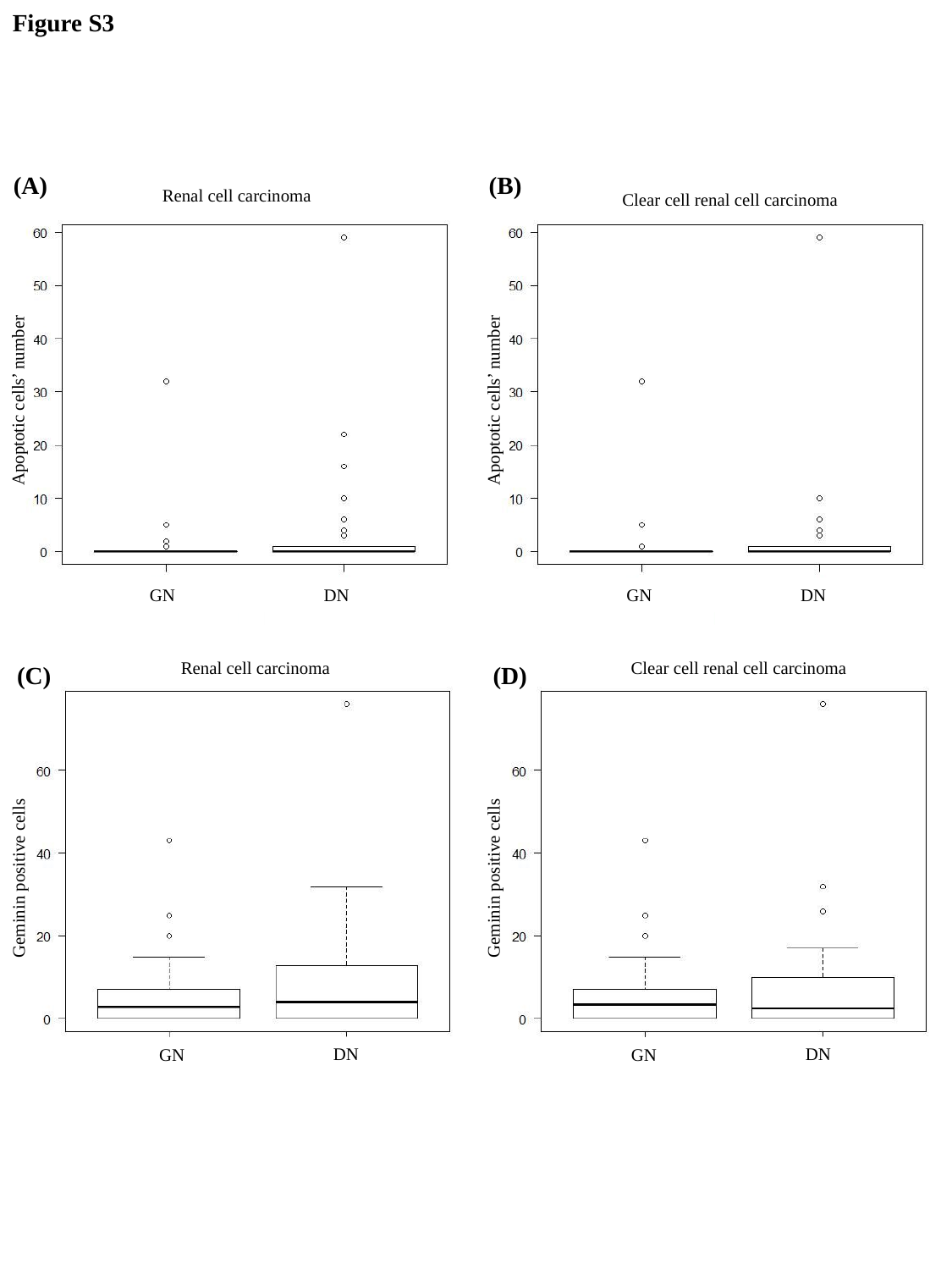

Figure S3
(B)
(A)
Renal cell carcinoma
Clear cell renal cell carcinoma
Apoptotic cells’ number
Apoptotic cells’ number
GN
DN
GN
DN
Renal cell carcinoma
Clear cell renal cell carcinoma
(C)
(D)
Geminin positive cells
Geminin positive cells
DN
DN
GN
GN
