# Supplementary material for: Dirty necrosis in renal cell carcinoma is associated with NETosis and systemic inflammation
Source: Cancer Med. 2022 Sep 20;12(4):4557–67. doi: 10.1002/cam4.5249 (PMC9972113; doi:10.1002/cam4.5249)
Supplement: Supplementary file 8 — Figure S4 [file CAM4-12-4557-s005.pptx]

## Slide 1
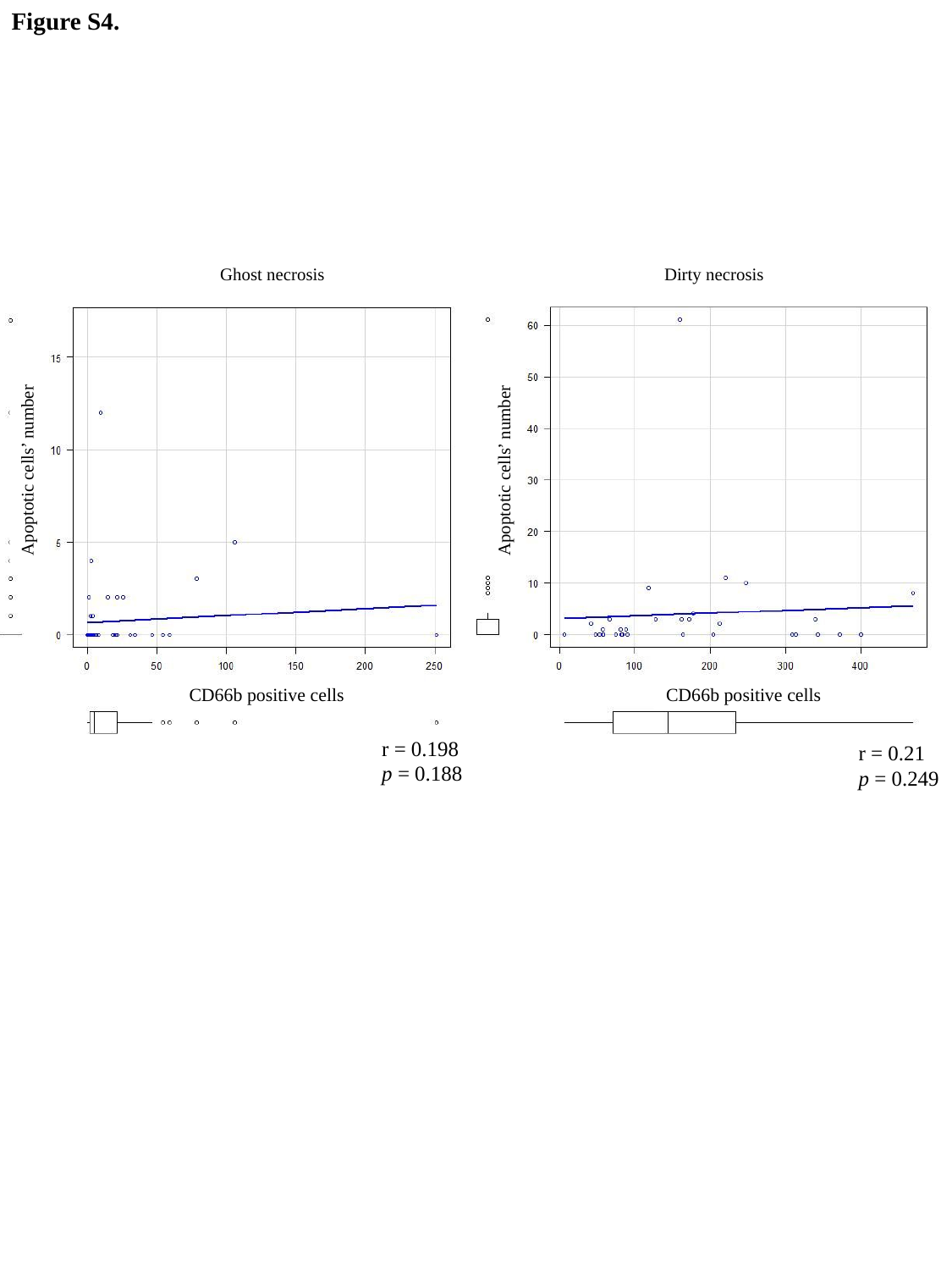

Figure S4.
Dirty necrosis
Apoptotic cells’ number
CD66b positive cells
 r = 0.21
 p = 0.249
Ghost necrosis
Apoptotic cells’ number
CD66b positive cells
 r = 0.198
 p = 0.188
